# O014. The diagnostic mistake: when the patient reports pain affecting eyes and benzodiazepines abuse without any glaucoma or any apparent organic cause

**DOI:** 10.1186/1129-2377-16-S1-A168

**Published:** 2015-09-28

**Authors:** Maria Nicolodi, Olivier D'Angri

**Affiliations:** Foundation Prevention and Therapy of Primary Pain and Headache, Florence, Italy

## Background

It is not so infrequent that a patient reports severe pain with a clear focus in/around the eye that looks like an atypical facial pain/persistent idiopathic facial pain. All the patients fulfilled DMS-IV criteria for depression or bipolar disorder-I and sleep and benzodiazepines overuse were reported as the only escape and cure treatment. This may or may not appear as a psychological flight reaction characterized by vegetative signs [1], or a medication-overuse headache. The diagnosis could be wrong even though the IHS/IASP and psychological criteria were respected. What could be the problem? We did not take into account that demodex is present even in man. Demodex -type A and type B-, the most serious non-neoplastic dermatological disease [2, 3], is not so widely known in human pathology [4]. This ascaris provokes discomfort and pain, the severity of which depends on the extent and seriousness of the disease [2–4], as well as on the pain proneness evidenced in third hyperalgesia test we proved several years ago [5]. Thus, pain proneness and pain redundancy might be present in both migraine sufferers and in their relatives.

## Aim

To evidence possible causes of therapeutic mistakes in persistent facial pain, chiefly affecting the eye area.

## Materials and methods

Observation started 26 years ago. Recruited patients (53 males; mean age 33.9 years ± 7.5 SD) suffering from atypical facial pain chiefly affecting the area of the eyes, were previously treated with all the substances commonly used in such a pain, namely tricyclics, negative modulators of excitatory aminoacids, selective serotonin reuptake inhibitors and norepinephrine reuptake inhibitors given as 3-month treatments and narcotics given as hospital short-lasting (5 days) regimen. Neither indomethacin 50 mg nor sumatriptan 6 mg, parenterally administered, induced relief.

## Results

None of the patients, except one, achieved pain relief, i.e. decrease vs baseline 17% on VAS 0-10. Only narcotics induced a benefit, which vanished when treatment was discontinued. Magnifiers observation focusing cilia showed that all non-responder patients were affected by demodex as evidenced with the use of a magnifying glass. The specific treatment for curing demodex completely relieved non-responders’ pain. Later (i.e., 3-25 years; mean 19.3 years+10.9 SD), episodic migraine without aura appeared in 25 patients.

## Conclusions

a) When a disease is rare it does not mean it can be neglected; b) an inherited abnormality of the central nervous system, namely inheritable hyperalgesia pattern seemingly provokes a redundancy of painful expression that may lead to diagnostic mistakes.

Written informed consent to publish was obtained from the patient(s).
